# Fecal microbiota and genetics in pediatric-onset orofacial granulomatosis and Crohn´s disease

**DOI:** 10.1038/s41598-025-90243-5

**Published:** 2025-02-19

**Authors:** Miikka Höyhtyä, Anu Haaramo, Anne Nikkonen, Rebecka Ventin-Holmberg, Nitin Agrawal, Jarmo Ritari, Brandon Hickman, Jukka Partanen, Heikki Alapulli, Jetta Tuokkola, Anne Salonen, Willem M de Vos, Kaija-Leena Kolho

**Affiliations:** 1 Faculty of Medicine, University of Helsinki, Helsinki, Finland; 2 Department of Otorhinolaryngology, Head and Neck Surgery, Helsinki University Hospital HUS and University of Helsinki, Helsinki, Finland; 3 Children´S Hospital, University of Helsinki and HUS, Helsinki, Finland; 4 Human Microbiome Research Program, Faculty of Medicine, University of Helsinki, Helsinki, Finland; 5 Folkhälsan Research Center, Helsinki, Finland; 6 Fin-HIT Research Group, Folkhälsan Research Center, Department of Public Health, Helsinki, Finland; 7 Finnish Red Cross Blood Service, Helsinki, Finland; 8 Department of Oral and Maxillofacial Diseases, Faculty of Medicine, University of Helsinki and Helsinki University Hospital HUS, Helsinki, Finland; 9 Clinical Nutrition Unit, Internal Medicine and Rehabilitation, Institute of Public Health, University of Helsinki and Helsinki University Hospital, Helsinki, Finland; 10 Clinical Nutrition, Kuopio and Department of Medicine, Endocrinology and Clinical Nutrition, University of Eastern Finland, Kuopio University Hospital, Kuopio, Finland; 11 Laboratory of Microbiology, Wageningen University, Wageningen, the Netherlands

**Keywords:** Children, Genotype, Inflammatory bowel diseases, Oral, Microbiology, Dysbiosis, Inflammatory bowel disease, Crohn's disease

## Abstract

Orofacial granulomatosis (OFG) is a rare chronic inflammatory condition. It is under debate, whether it is a condition of its own or merely a subtype of Crohn’s disease (CD). We aimed to search for markers characteristic of patients with pediatric-onset OFG compared to patients with pediatric-onset CD. We recruited young patients with OFG (with or without CD, *n* = 29), CD (*n* = 24), and healthy controls (*n* = 20). All participants provided a fecal sample for microbiota and calprotectin analyses and saliva for DNA analysis of genes associated with OFG and kept a 3-day food diary. Oral disease activity was evaluated using The Oral Disease Activity Score by an otorhinolaryngologist and a dentist. We observed decreased relative abundance in class Clostridia and increased relative abundances of classes Actinobacteria and Bacilli in the feces of patients with OFG when compared to patients with CD and healthy controls. The relative abundances of *Bifidobacterium adolescentis* increased and *Faecalibacterium prausnitzii* decreased along with the increase in the Oral Disease Activity Score. We found the NOD2 gene rs8057341 allele A to be enriched in patients with OFG compared to patients with CD. These findings support the theory that OFG is a distinct disease phenotype.

**Supplementary Information:**

The online version contains supplementary material available at 10.1038/s41598-025-90243-5.

## Introduction

Orofacial granulomatosis (OFG) is a rare inflammatory condition, characterized by noncaseating granulomatous inflammation in the orofacial area^1^. Some consider it an umbrella term, encompassing the oral manifestations of several systemic diseases with granulomatous inflammation, such as Crohn’s disease (CD), sarcoidosis, granulomatosis with polyangiitis, and Melkersson-Rosenthal syndrome^1^. Also, OFG may be idiopathic, localizing only in the oral mucosa and face^2^.

The symptoms of OFG may vary, including lip swelling, oral ulcerations, gingival overgrowth, angular cheilitis, vertical fissures of the lips, and cobblestone-like mucosa of the mouth^3,4^. The exact pathogenesis of OFG remains unclear. Genetic, allergic, immunologic, and infectious mechanisms have been proposed^3^. Due to the large heterogeneity of the disease group, and the relative rarity of the disease, the results are variable. The diagnosis of OFG is based on patient history and typical clinical features^3^. Biopsy is not mandatory and can be negative for granulomas and indistinguishable from mouth lesions in CD^1,3,5^. A study, comparing patients with OFG and patients with concomitant CD and OFG, found that patients with only OFG had a higher number of CD3-expressing T cells and more CD11c-expressing dendritic cells in the mouth lesions^6^. Also, patients with OFG have been reported to have subepithelial B cells, with IgE expression in the oral mucosa^7^. This finding suggests an IgE-mediated mechanism.

The role of genetic factors behind OFG is not fully understood. Gibson et al. reported an association between OFG and HLA serotypes A3, B7, and DR2 ^8^. Another study showed an association between HLA B*44 and OFG + CD when compared to patients with only CD^9^. However, there was no statistically significant difference, when comparing OFG + CD to healthy controls^9^. A study by Mentzer et al. reported that NOD2 variant p.L1007insC was associated with OFG + CD and IL23R variant p.R381Q with all OFG when compared with controls^10^. Currently, there is no available genetic marker for OFG^3^.

A recent review encompassing 14 studies and 507 patients summarized the association between CD and OFG^4^. This association was more prominent in pediatric-onset OFG^4^. In another review on pediatric OFG and CD, up to 40% of patients with OFG had concomitant CD, and OFG anticipated the occurrence of CD^11^. Due to similarities between OFG and CD in histopathology and the high concomitancy of these conditions, it is under debate whether OFG is a condition of its own, or merely a subtype of CD^3,11^.

A recent study on the relation of oral microbiota and OFG discovered decreased levels of *Streptococcus salivarius*in patients with CD or OFG in comparison to healthy controls. In addition, there were some minor differences in less prominent taxa^12^. To our knowledge, no studies regarding the fecal microbiota in OFG have been made so far.

We studied the fecal microbiota and genetics in pediatric-onset OFG in comparison to patients with pediatric-onset CD without oral involvement and healthy controls. We hypothesized that the oral involvement would be reflected in the fecal microbiota and aimed to find genetic variants to associate with OFG. Our previous study examined HLA-genotypes on the same dataset^9^. Based on previous literature, we prospectively chose to study whether variants of genes FUT2, NOD2, BTNL2, or IL23R would correlate with OFG(+ CD) compared to patients with CD^10^. An improved understanding of the fecal microbiota and genes in OFG would further help us understand the unknown underlying mechanisms of OFG in pediatric-onset disease.

## Results

### Patient characteristics

We included 29 patients with OFG of which 6 (21%) had no signs of intestinal disease, the rest were also diagnosed with CD. As comparison groups, we analyzed 24 patients with pediatric-onset CD and 20 healthy controls. Patient characteristics are shown in Table 1 and medications and results of laboratory investigations in Table 2. According to fecal calprotectin, there was no difference in the activity of the CD between patients with OFG and mere CD (Table 2).

**Table 1:** Patient characteristics.

	OFG (*n* = 29)	CD (*n* = 24)	HC (*n* = 20)	*p*-value OFG /CD	*p*-value OFG / HC	*p*-value CD / HC
Males (%)	22 (76)	14 (58)	10 (50)	0.240	0.075	0.762
Median age (range)	14.4 (6.7–31)	22.0 (19.3–27.5)	21.8 (18.9–28.9)	**< 0.001**	**<0.001**	1.00
Median age (range) at CD diagnosis	12.3 (1.9–17.6)	13.2 (8.1–16.4)	NA	0.67	NA	NA
Median age (range) at OFG diagnosis	12.3 (2.9–15.8)	NA	NA	NA	NA	NA
Biopsy confirmed OFG (%)	13 (45)	NA	NA	NA		
Diagnosis of CD (%)	21 (72)	24 (100)	NA	NA	NA	NA
**Paris Classification of CD** -L1 -L2 -L3 -L4a or L4b	4 5 10 2	2 4 17 3 (2 with coexisting L3)	NA NA NA NA	0.396 0.713 0.594 1	NA NA NA NA	NA NA NA NA
Abdominal surgery; N (%)	3 (10)	4 (17)	0 (0)	0.321	0.26	0.074
Median Oral Disease Activity Score (range)	10 (0–33)	0 (0–3)	0 (0–1)	**< 0.001**	NA	NA
Smoking (%)	1 (3.4)	0 (0)	2 (10)	1	0.559	0.201
Food allergies (%)	9 (31)	4 (17)	2 (10)	0.338	0.162	0.673
Avoidance diet (%)	18 (62)	6 (25)	2 (10)	**0.005**	**< 0.001**	0.26
Atopy (%)	6 (21)	4 (17)	0 (0)	1	0.069	0.114
Dietary fiber, % of daily recommendation (range)*	50.7 (17.9–107)	58.4 (29.1–137.4)	65.6 (23.2–156)	0.059	**0.02**	0.685

* daily fiber recommendation ≥ 25–35 g^21^.

CD, Crohn’s disease; OFG, Orofacial granulomatosis; HC, healthy controls; L1, Distal 1/3 ileal ± limited cecal disease; L2, Colonic; L3, Ileocolonic; L4a, Upper disease proximal to the ligament of Treitz; L4b, Upper disease distal to the ligament of Treitz and proximal to distal 1/3 ileum.

**Table 2:** Medication and laboratory tests at the time of obtaining fecal samples for microbiota analyses.

	OFG (*n* = 29)	CD (*n* = 24)	HC (*n* = 20)		*p*-value OFG /CD	*p*-value OFG / HC	*p*-value CD / HC
**Medication** -Oral glucocorticoids (%) −5-ASA (%) -Methotrexate (%) -Azathioprine -Anti-TNF-α (%) -Other biologics (%)	2 (6.9) 11 (38) 2 (6.9) 4 (14) 3 (10) 0 (0)	1 (4.2) 5 (21) 1 (4.2) 11 (46) 11 (46) 1 (4.2)	0 (0) 1 (5) 0 (0) 0 (0) 1 (5) 0 (0)	1 0.126 1 **0.015** **0.005** 0.453		NA NA NA NA NA NA	NA NA NA NA NA NA
Antibiotics 6 months prior (%)	11 (38)	2 (8.3)	2 (10)	**0.023**		**0.047**	1.00
Laboratory tests, median (range) - fecal calprotectin, µg/g - CRP, mg/l - ESR, mm/h - Hemoglobin, g/l - Albumin, g/l	183 (6–1833) 6 (2–9) 8 (2–38) 138 (92–160) 41 (36–41)	100 (6–2488) 5 (4–13) 8 (2–39) 141 (106–167) 36.5 (35–42)	19.5 (5–174) 19 (14–67) 2 (2–66) 141 (125–166) NA	0.54 0.82 0.057 0.31 0.058		**0.001** **0.027** **0.017** 0.25 NA	**0.01** **0.013** 0.088 0.85 NA
No intestinal inflammation (fecal calprotectin < 100 µg/g)	12 (41)	11 (46)	18 (90)	0.11		**< 0.001**	**0.002**

CD, Crohn’s disease; OFG, Orofacial granulomatosis; HC, healthy controls; 5-ASA, 5-aminosalicylic acid; Anti-TNF-α, Anti-tumor necrosis factor α; CRP, C-reactive protein; ESR, erythrocyte sedimentation rate.

### Fecal microbiota between patients with OFG, patients with CD, and healthy controls

**Fig. 1:**
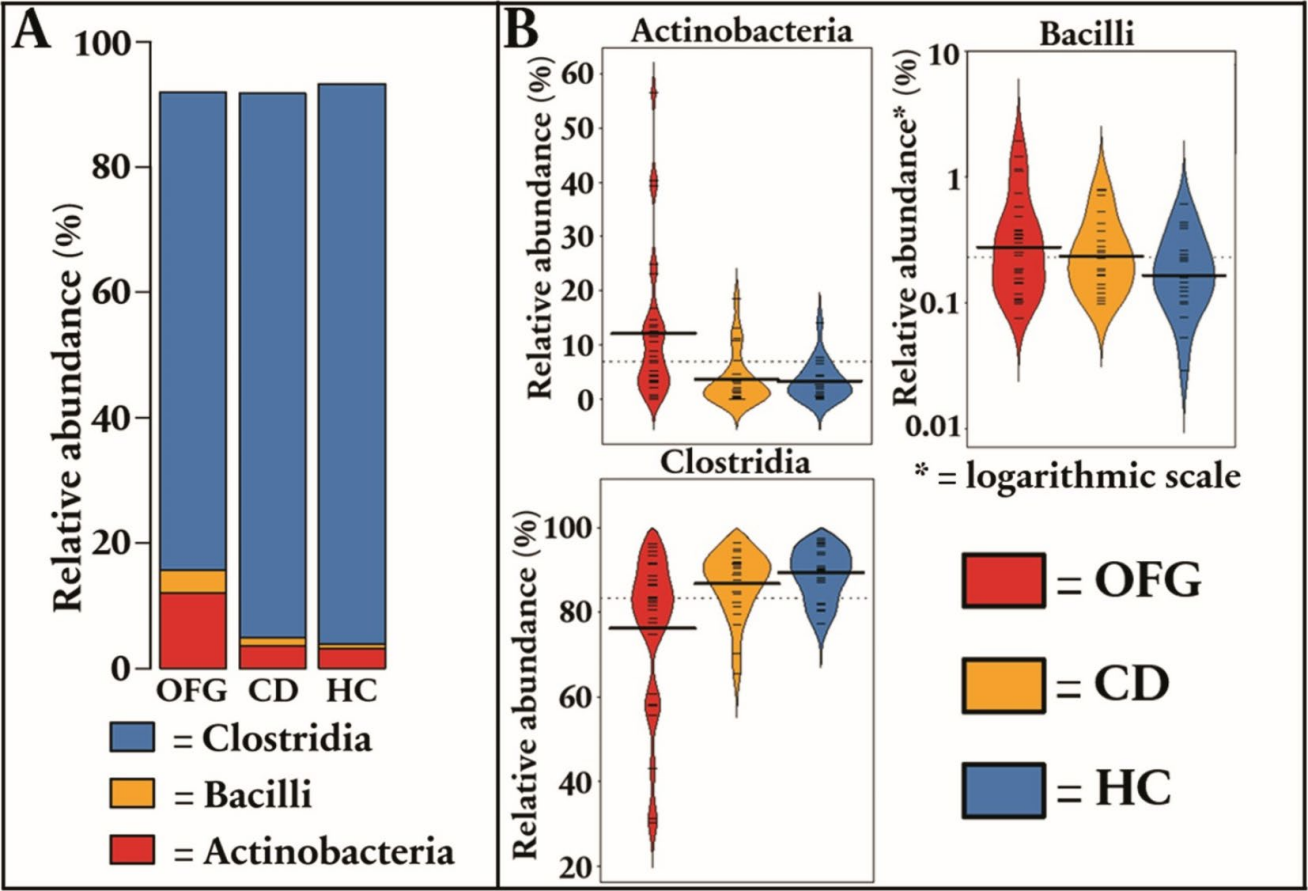
**(a)** Stacked plot regarding class-level differences in fecal microbiota between patients with pediatric-onset orofacial granulomatosis (OFG), patients with pediatric-onset Crohn´s disease (CD) and healthy controls **(b)** Violin plots show statistically significant class-level differences between patients with OFG, patients with CD and healthy controls.

When compared to patients with CD and healthy controls, the feces of patients with OFG had an increased relative abundance of phylum Actinobacteria (by 3.64-fold, p = *0.008* and by 3.44-fold, p = *0.008*) and class Bacilli (by 2.29-fold, p = *0.035* and by 3.48-fold, p = *0.003*), but a reduced relative abundance of class Clostridia (1.08, p = *0.008* and 1.14, p < *0.001*), respectively (Fig. 1).

**Fig. 2:**
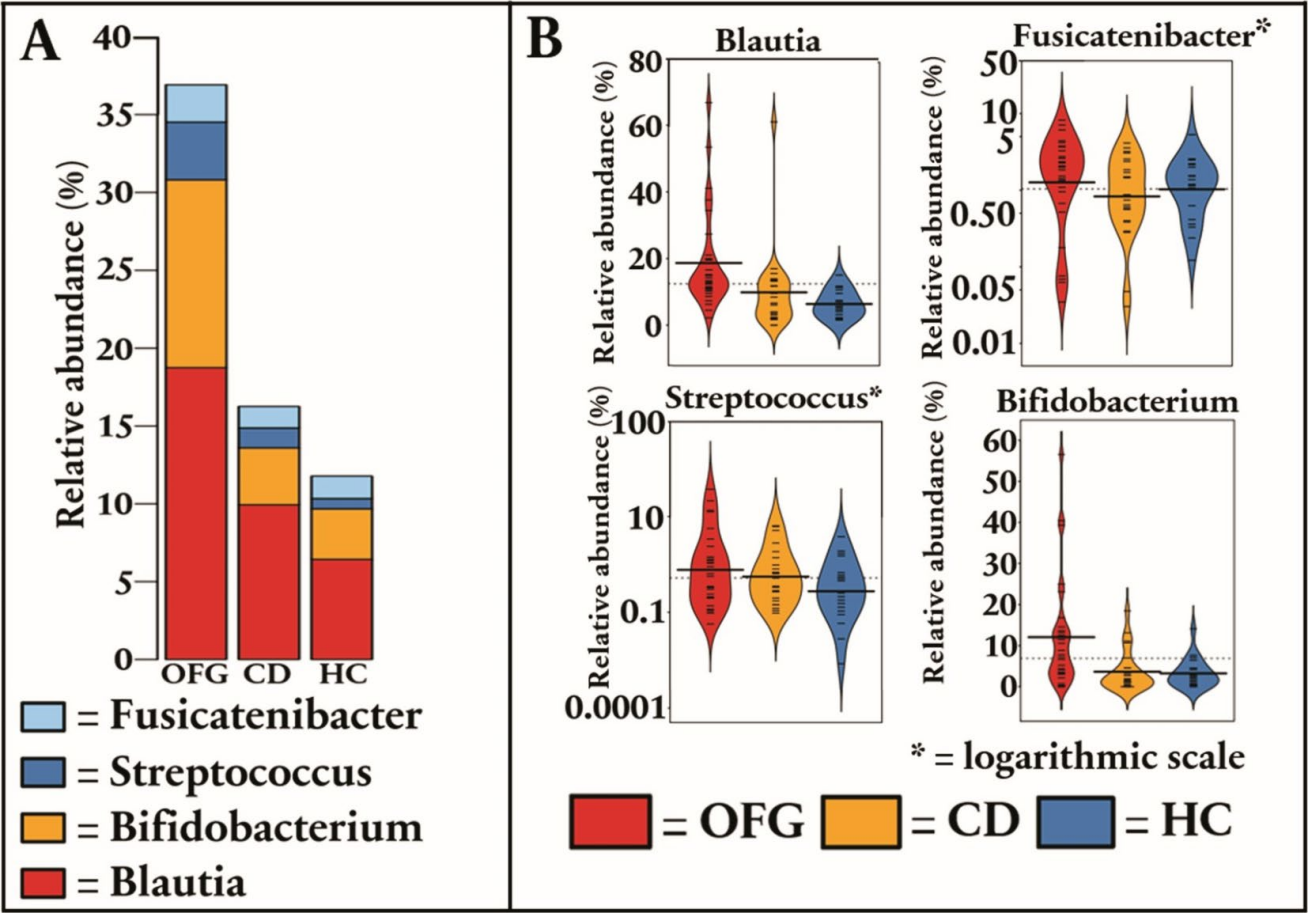
**(a)** Stacked plot regarding genus-level differences in fecal microbiota between patients with pediatric-onset orofacial granulomatosis (OFG), patients with pediatric-onset Crohn´s disease (CD) and healthy controls **(b)** Violin plots show statistically significant genus-level differences between patients with OFG, patients with CD and healthy controls.

At the genus level, patients with OFG had an increased relative abundance of *Bifidobacterium* (by 3.64-fold, p = *0.008* and by 3.44-fold p = *0.008*), *Streptococcus* (by 2.39-fold, *p* = 0.035 and by 3.49-fold, p = *0.003*), *Blautia* (by 2.47-fold, p = *0.003* and by 3.05-fold, p < *0.001*), *Fusicatenibacter* (by 2.73-fold, p = *0.003* and by 2.64-fold, p = *0.003*), when compared to patients with CD and healthy controls, respectively. Also, patients with OFG had a reduced relative abundance of genera *Kineothrix* (by 1.98-fold, p = *0.003*) *Coprococcus* (by 5.0-fold, p = *0.003*), and *Faecalibacterium* (by 2.15-fold, p = *0.005*) when compared to healthy controls (Fig. 2). All statistically significant differences of other taxonomic levels are listed in Supplementary Table 1.

We also tested whether age influenced the fecal microbiota. Along increasing age, the relative abundance of Ruminococcaceae (slope 0.02, FDR *p* < 0.0001) and genera *Faecalibacterium* (slope 0.014, *p FDR < 0.001*), *Gemminger* (slope 0.034 FDR p < *0.001*) increased.

## Fecal microbiota and oral symptom severity

In all patients, orofacial findings were recorded using the Oral Disease Activity Score chart for OFG^13^. Patients with OFG had higher Oral Disease Activity Scores than patients with CD (medians 10 (range 0–33) and 0 (range 0–3), *p* < 0.0001, respectively). We tested whether the Oral Disease Activity Score was associated with the composition of the fecal microbiota of OFG and CD patients. We found the relative abundance of species *Bifidobacterium adolescentis* (slope 0.03, FDR p < *0.001*), *Dorea longicatena* (slope 0.02, FDR p < *0.001* ), *Agathobaculum butyriproducens* (slope 0.029, FDR p < *0.001*) to increase as Oral Disease Activity Score increased, whereas the relative abundance of *Faecalibacterium prausnitzii* (slope − 0.026, FDR p < *0.001*) and *Gemminger formicilis* (slope − 0.027, FDR p < *0.001*) decreased when the Oral Disease Activity Score increased. There was no significant correlation between fecal calprotectin and the Oral Disease Activity Score (Spearman correlation coefficient 0.154, *p* = 0.28).

## Data on nutrition and food allergies

There was a significant difference (*p* = 0.02) in dietary fiber gain between patients with OFG and healthy controls (Table 1). The fiber intake in most patients was lower than recommended (Table 1). However, no significant association between dietary fiber intake and fecal microbiota composition was seen.

Patients with OFG did not report food allergies more frequently compared to patients with CD or healthy controls (Table 1). However, elimination diets (due to gastrointestinal or mouth symptoms) were more common in patients with OFG compared to patients with CD and healthy controls (62%, 25%, and 10% respectively (Table 1). Most avoided food products by OFG patients were varying vegetables (31%) and fruits (17%), chocolate (14%), dairy products (14%), carbonated drinks (12.5%), vinegar and pickled products (12.5%), cinnamon (7%), and strongly flavored food (7%). Most patients with self-reported food allergies had multiple allergies, and most patients with an elimination diet avoided multiple food products (Supplementary Table 2).

### Genetic analyses

All patients with OFG and 22 patients with CD provided a saliva sample for DNA analysis. In two patients with OFG and two with CD, the quantity of DNA allowed only FUT2 genotyping. The original genotype data contained 34, 38, 42, and 69 SNPs in the target genes IL23R, NOD2, FUT2, and BTNL2, respectively. Out of these, 28, 18, 10, and 43 SNPs, respectively, could be analyzed for association due to limitations in allele frequency and sample size. The top result was obtained for NOD2 gene rs8057341 allele A with a p-value of 0.012 and OR 4.10 (95% CI: 1.36–12.30) when comparing patients with OFG to patients with CD. The FUT2 gene non-secretor phenotype was not statistically different between any of the study groups (17% of OFG, 25% with CD, and 10% of healthy controls, *p* = 0.43).

We then examined the association of NOD2 gene rs8057341 allele A on the fecal microbiota in our study population. When comparing patients with no allele A to patients with one allele A (heterozygotes), the relative abundance of genus *Faecalibacterium* was increased in heterozygotes (by 1.1-fold, FDR p < *0.001*). When comparing patients with two rs8057341 A alleles (homozygotes) to patients without allele A, the homozygotes had increased relative abundance of *Faecalibacterium* (by 1.73-fold, FDR p < *0.0001*), but reduced abundance of *Bifidobacterium adolescentis* (by 319-fold, FDR p < *0.001*) and *Blautia hominis* (by 5.9-fold, FDR p = *0.009*).

## Discussion

We searched for markers that were associated with pediatric-onset OFG (with or without CD) by examining the fecal microbiota and genetics and compared the findings to patients with sole pediatric-onset CD and healthy controls. To our knowledge, this is the first study to examine fecal microbiota in OFG. We also tested how disease activity was reflected in fecal microbiota, examining fecal calprotectin and oral symptoms systematically at the time of the fecal sample.

Fecal microbiota differed significantly between patients with pediatric-onset OFG, CD, and healthy controls. Interestingly, this difference was seen in high taxonomic levels, where patients with OFG had a reduction in class Clostridia and an increased relative abundance of phylum Actinobacteria and class Bacilli. Moreover, the fecal microbiota in patients with OFG deviated more from healthy controls than the microbiota of patients with CD, which is interesting considering that most patients with OFG also had CD. The level of intestinal inflammation according to fecal calprotectin was similar between OFG and CD groups and thus does not explain the more aberrant microbiota in OFG (+ CD) compared to CD.

Comparable findings were seen at the genus level, where patients with OFG had an increased relative abundance of *Bifidobacterium*, *Streptococcus*,* Blautia*, and *Fucicatenibacter* when compared to both patients with CD and healthy controls. Previously, an increased relative abundance of *Streptococcus*has been associated with pediatric CD, when compared to healthy controls^14,15^. However, reduced relative abundances of *Blautia* and *Bifidobacterium *have been reported in pediatric IBD and pediatric CD compared to healthy controls^14,16^. Therefore, it is surprising that patients with OFG (most with concurrent CD) had increased relative abundances of *Blautia* and *Bifidobacterium*. The unique microbiota, which is even more aberrant in OFG than in CD suggests that OFG is indeed a unique disease entity.

When examining the relation of the Oral Symptom Activity score and fecal microbiota, the relative abundance of *Bifidobacterium adolescentis* increased whereas the relative abundance of *Faecalibacterium prausnitzii* decreased along the more active oral disease. Previously, a lower relative abundance of *Bifidobacterium adolescentis* has been reported in ulcerative colitis, and *Bifidobacterium adolescentis *supplementation stimulated Th2/Treg response and remodeled the gut microbiota in DSS-induced chronic colitis rats^17^. *Faecalibacterium prausnitzii *is a known anti-inflammatory butyrate producer and lower relative abundances have been reported in IBD^14^. In our data, patients with more extensive mouth disease also had fecal microbial signatures pointing toward inflammation. This is particularly interesting, since intestinal inflammation, tested by fecal calprotectin, did not associate with mouth symptom score. The possible connection between inflammatory taxa in the gut and oral symptoms is further supported by the notion that OFG treatment options, such as dietary restrictions and anti-inflammatory medication, also modify the fecal microbiota and may increase anti-inflammatory taxa^18,19^.

In feces, the relative abundance of *Faecalibacterium* was reduced when comparing patients with OFG to healthy controls. However, *Faecalibacterium* did not differ statistically significantly between patients with CD and OFG. *Faecalibacterium* also increased along with increasing age, and OFG patients were younger than healthy controls. This could confound the result. However, age does not explain why there was no difference between patients with OFG and patients with CD. Previously, low relative or absolute abundances of *Faecalibacterium *have been reported when patients with CD were compared to healthy controls^14,15^. In our study, most patients with CD had mild disease activity according to fecal calprotectin. Also, most patients with OFG had concomitant CD. These factors could explain the similar level of *Faecalibacterium*, and the difference to healthy controls.

### Nutrition

The patients with OFG had lower dietary fiber gain than healthy controls according to a 3-day food diary at the time of the study. However, this was not reflected in the microbiota. This is probably due to small differences in fiber gain between groups which would need a larger sample size for statistical power. The fiber intake in most patients was lower than recommended, which is in line with reports from the Finnish population^20,21^.

### Genetic markers

We observed the NOD2 gene variant, rs8057341 allele A, to be more frequent in patients with OFG when compared to patients with CD. Upon activation, the NOD2 gene produces proteins that modulate the transcription of genes encoding proinflammatory cytokines IL-8, TNF-α, and IL-1β^22^. Previously, NOD2 gene variants have been associated with an increased risk for adult and pediatric CD^22^. Intriguingly, a previous study on OFG found NOD2 gene variant p.L1007insC to be enriched in patients with concomitant CD and OFG but not in patients with only OFG^10^. Our findings support the previous findings that there are differences in the genetic background between CD and OFG^8–10^. Not only did the NOD2 gene rs8057341 allele A differ between patient groups, but it was also associated with changes in the fecal microbiota. Patients with two rs8057341 A alleles had reduced abundance of *Faecalibacterium*, but also significantly reduced relative abundance of *Bifidobacterium adolescentis.* Since our sample size from patients was limited for genetic analyses, our results are considered preliminary.

In addition to NOD2 variants, we analyzed as well other genetic variants associated with IBD such as FUT2-gene non-secretor genotype^23^. Interestingly, the non-secretor genotype was previously associated with lower gut microbiota diversity, richness, and lower abundance of *Bifidobacterium *in the first months of life^23^. However, some studies have found no association with gut microbiota and FUT2 genotypes^23^. In our samples there were no differences in FUT2 gene secretor genotypes between patient groups, the average frequency of non-secretors being close to 20% as reported from another Finnish patient cohort with IBD^24^.

Atopy was reported in 21% of OFG patients, reflecting the prevalence in the adult Finnish population^25^. Self-reported adverse reactions to food were not more frequent in patients with OFG. However, avoiding certain food products was more common in patients with OFG (18%) compared to patients with CD and healthy controls (6% and 2% respectively). The most avoided food ingredients were various vegetables and fruits, chocolate, dairy products, carbonated drinks, vinegar, and pickled products. As expected, with multiple avoidance diets, we found no common allergens to be enriched in the diet of the OFG patients. Previously, up to 80% of patients with OFG were reported to have an IgE-mediated allergy^26^. Several studies have reported hypersensitivity to food products such as dairy products, chocolates, cocoa, eggs, peanuts, monosodium glutamate, and carmoisine in patients with OFG^3^. This is supported by reports in which the exclusion diet has been successful in treating the disease^26^. These findings point out that there is a subgroup of patients, in which adverse reactions to food play a role. However, it is not certain whether such reactions are causative or merely worsening the disease.

Most of our patients with OFG had concomitant CD. According to fecal calprotectin, there was no difference in the activity of the CD between patients with OFG and mere CD. Most patients received systemic treatment for CD, and approximately half of the patients with either OFG + CD or CD were in remission. Most patients not in remission had mild disease activity at the time of the study. All analyses were adjusted according to treatments with noted significant differences between groups, i.e., 5-ASA, azathioprine, and anti-TNFα and antibiotic treatment in the 6 months before sampling, to diminish the effect of these as confounders.

### Strengths and limitations

To our knowledge, we conducted the first study to examine the fecal microbiota, genetics, and association of oral disease activity to fecal microbiota in OFG. As a strength, the use of immunomodulatory medications and antibiotics was reported and accounted for in the microbiota analyses. The fecal samples were immediately put in a freezer. Also, all patients kept a 3-day food diary to detect the differences in diet when fecal samples were provided. As an additional strength, all oral findings were assessed and scored by a dentist and an otorhinolaryngologist. We had a relatively large cohort, considering the rarity of the pediatric-onset disease. On the other hand, due to sample size, we had to examine both patients with only OFG and patients with OFG and CD as one group. However, even with most OFG patients with simultaneous CD, the groups differed genetically and microbiologically. As a weakness, the patients with OFG were younger than the patients with CD and healthy controls. This might have confounded the results, especially regarding the lower relative abundance of *Faecalibacterium*in patients with OFG. However, we found no association of other differing bacteria between study groups and age in our dataset. Unfortunately, the saliva samples were not available for microbiota analyses. Also, we studied the relative abundances of the bacteria with no quantitative data that may give further insight into the composition of the microbiome^27^. Future studies regarding the differences between oral and fecal microbiota in active OFG and OFG in remission could highlight whether oral and fecal microbiota simultaneously change according to disease activity. This would further highlight the role of microbiota in OFG. Also, larger cohorts examining genetic risk variants in OFG are warranted, since our cohort was relatively small for genetic analyses and the findings are considered preliminary.

In conclusion, our study shows that the fecal microbiota and genetics differed between patients with OFG and patients with CD. These findings open the way for further research on the unique characteristics of OFG. An interesting discovery was that the activity of oral disease was reflected in the fecal microbiota. This raises questions about whether intestinal microbiota plays a larger yet unknown role in OFG.

## Methods

### Study population

We studied fecal microbiota in 29 patients with pediatric-onset OFG. The patients were recruited for a study examining oral and oropharyngeal manifestations during a study visit and the main findings were published (13 ). In brief, the diagnosis of OFG was based on typical clinical features, examined by a dentist and a gastroenterologist. In most patients, a mucosal sample showing granulomatous inflammation in the oral cavity was recorded. Notably, most patients (*n*= 24) had CD diagnosed according to the revised Porto criteria, including biopsies and imaging^28^. One patient with OFG was diagnosed with CD, 6.8 years after the study visit. For comparison group to study oral findings, we enrolled patients who were diagnosed with pediatric CD and enrolled healthy controls to these CD patients matched for age and sex (*n*= 20) from the Population Registration Centre. All participants in the comparison group were evaluated at a study visit as well by an otorhinolaryngologist, including fiberoscopic laryngoscopy, and a dentist as previously described^13,29^. The participants gave a fecal sample at home, which was immediately frozen at −20 ^o^C, and brought the frozen sample when attending a study visit where the sample was put in a freezer at −30 ^o^C and transferred to a freezer of −70 ^o^C until microbiota and calprotectin analyses. Additionally, we collected saliva samples for DNA analysis during the study visit^13^. Before the study visit, all the participants kept a 3-day food diary. Average daily fiber intake was calculated from the food diaries with software (AivoDiet, Aivo Finland Oy, Turku, Finland) which uses a national database of foods.

As reported earlier, when examined during the study visit, oral mucosa was the most common site of OFG findings (90% of patients), followed by lower lip (76%), gingival lesions (66%), and angular cheilitis (66%). The patients with concomitant OFG and CD had more severe orofacial findings than patients with sole OFG, with Oral Disease Activity total scores for orofacial findings being higher^13^. More detailed data on all the findings are available in our previous articles^13,29^.

We evaluated the degree of intestinal inflammation using fecal calprotectin as a marker of inflammation. A cut-off of < 100 µg/g defines an upper limit for normal values and remission^27^. The fecal calprotectin level was measured in a routine clinical laboratory using an ELISA kit from Calpro AS (Calpro/Calprolab, Oslo, Norway).

### Bacterial DNA extraction and analysis

Bacterial DNA was extracted from fecal samples using mechanical cell lysis that efficiently extracts bacterial community DNA^30–32^. Illumina MiSeq paired-end sequencing of the 16 S rRNA gene was performed according to the manual from Illumina (https://support.illumina.com/documents/documentation/chemistry_documentation/16s/16s-metagenomic-library-prep-guide-15044223-b.pdf). In brief, first primers targeting the bacterial hypervariable V3-V4 region (viite 32) were used to amplify the respective 16 S rRNA gene fragment. The size of the PCR product was expected to be ~ 640 base pairs (bp) and verified on a Bioanalyzer DNA 1000 chip (Agilent Technology, Santa Clara, CA, USA). The amplicons were in a subsequent PCR-reaction tagged with barcoded primers and after clean-up with AMPure XP beads (Beckman Coulter, Copenhagen, Denmark) and confirmation of the right size of the target, pooled. The pooled library was sequenced with an Illumina MiSeq instrument using paired end 2 × 300 bp reads and a MiSeq v3 reagent kit (600 cycles)^32^.

### Genetic analyses

The saliva samples were genotyped on the Illumina GSA platform as described previously^33^. Sample and phenotype data were managed with R software v4.2.2 ^34^ using packages *tidyverse* v1.3.1 ^35^ and *readxl* v1.3.1 ^36^. The target gene names were mapped to Ensembl gene IDs using the Ensembl genome browser (https://www.ensembl.org/index.html). The target gene positions for the human genome build GRCh37 were retrieved from the Ensembl database using the R package *biomaRt* v2.50.1 ^37,38^ with parameters host=”https://grch37.ensembl.org”, and path=”/biomart/martservice” by using the Ensembl gene IDs as inputs. SNP IDs within the target genes were identified based on the Ensembl gene coordinates and the SNP genotypes were extracted from the full genotype data using plink2 v2.00a3LM^39,40^. Association analysis for the extracted SNPs in an OFG vs. CD case-control setting was performed with plink2 generalized linear model command glm with default parameters except allow no covariates and ci 0.95. FUT2 genotyping was performed separately^41^.

### Statistical analysis

Analysis of the 16 S rRNA gene amplicon sequence data was conducted in R, using the package *mare*^42^. Pre-processing, quality filtering, and taxonomic annotation of the reads were performed using USEARCH^43^by mapping the reads to the SILVA 16 S rRNA reference database version 115^42^, restricted to gut-associated taxa (available through the R package *mare*^44^. Only high-quality forward reads trimmed to 150 nucleotides were used for analysis. Comparative analyses for the differences in the relative abundances of common bacteria at different taxonomic levels were performed with *mare* functions “GroupTest”, “CovariateTest” and “ChangeTest” which implements generalized linear models using negative binomial distribution from *MASS*^45^, with tools from packages *vegan*^46^, *MASS*^45^, and *nlme*^47^.

All patients with OFG (with or without CD) were grouped for statistical power. All categorical variables between study groups with value < 5 were tested for statistical significance using Fisher’s exact test and all other variables using Pearson’s chi-squared test (Table 1). All numerical variables were non-normally distributed and were tested for statistical significance using the Kruskal-Wallis test. When comparing two continuous variables, we used Spearman’s rank correlation coefficient. P-values for taxon-specific differences in fecal microbiota were corrected for false discovery rate (FDR; Benjamini–Hochberg).

We tested for factors associated with the fecal microbiota using principal coordinates analysis (PCoA). As confounders in microbiota analysis, we chose 5 of the most influential factors in microbiota composition based on the statistically significant difference between the study groups and our previous work^27^. These five confounders comprised abdominal surgery, antibiotic treatment within 6 months before sampling, and the key medications: 5-ASA, azathioprine, and anti-TNFα.

### Ethical considerations

The study was approved by the Ethics Committee of Helsinki University Hospital on 18.9.2013 and the supplementary approval on 13.7.2016 (diary number 240/13/03/02/13 for both). The patients and/or their guardians signed an informed consent form. All healthy controls and/or their guardians also signed an informed consent form. The study was conducted in accordance with the Declaration of Helsinki.

CD, Crohn’s disease; OFG, Orofacial granulomatosis; HC, healthy controls; 5-ASA, 5-aminosalicylic acid; Anti-TNF-α, Anti-tumor necrosis factor α; CRP, C-reactive protein; ESR, erythrocyte sedimentation rate.

## Electronic supplementary material

Below is the link to the electronic supplementary material.

Supplementary Material 1

Supplementary Material 2

## Data Availability

Sequence data that support the findings of this study have been deposited in the European Nucleotide Archive with the primary accession code PRJEB84421 (https://www.ebi.ac.uk/ena/browser/view/PRJEB84421).
